# Antifungal Activity of Cyclic Tetrapeptide from *Bacillus velezensis* CE 100 against Plant Pathogen *Colletotrichum gloeosporioides*

**DOI:** 10.3390/pathogens10020209

**Published:** 2021-02-15

**Authors:** Vantha Choub, Chaw Ei Htwe Maung, Sang-Jae Won, Jae-Hyun Moon, Kil Yong Kim, Yeon Soo Han, Jeong-Yong Cho, Young Sang Ahn

**Affiliations:** 1 Department of Forest Resources, College of Agriculture and Life Sciences, Chonnam National University, Gwangju 61186, Korea; vanthachoub@gmail.com (V.C.); lazyno@naver.com (S.-J.W.); mjh132577@naver.com (J.-H.M.); 2 Division of Agricultural and Biological Chemistry, Institute of Environmentally Friendly Agriculture, College of Agriculture and Life Sciences, Chonnam National University, Gwangju 61186, Korea; chaweihtwemaung@gmail.com (C.E.H.M.); kimkil@jnu.ac.kr (K.Y.K.); 3 Division of Plant Biotechnology, Institute of Environmentally Friendly Agriculture, College of Agriculture and Life Sciences, Chonnam National University, Gwangju 61186, Korea; hanys@jnu.ac.kr; 4 Department of Food Science and Technology, College of Agriculture and Life Sciences, Chonnam National University, Gwangju 61186, Korea

**Keywords:** antagonistic bacteria, antifungal cyclic tetrapeptide, anthracnose disease, mycelial growth, spore germination, biocontrol agent

## Abstract

The aim of this study was to investigate the antifungal activity of a cyclic tetrapeptide from *Bacillus velezensis* CE 100 against anthracnose-causing fungal pathogen *Colletotrichum gloeosporioides*. Antifungal compound produced by *B. velezensis* CE 100 was isolated and purified from ethyl acetate extract of *B. velezensis* CE 100 culture broth using octadecylsilane column chromatography. The purified compound was identified as cyclo-(prolyl-valyl-alanyl-isoleucyl) based on mass spectrometer and nuclear magnetic resonance analyses. This is the first report of the isolation of a cyclic tetrapeptide from *B. velezensis* CE 100 culture filtrate. Cyclic tetrapeptide displayed strong antifungal activity at concentration of 1000 µg/mL against *C. gloeosporioides* mycelial growth and spore germination. Our results demonstrate that the antifungal cyclic tetrapeptide from *B. velezensis* CE 100 has potential in bioprotection against anthracnose disease of plants caused by *C. gloeosporioides*.

## 1. Introduction

Phytopathogenic fungi are serious threats to crops. They can reduce the quality and yield of agricultural products [1,2]. Generally, the ideal way to prevent fungal invasion is by the application of fungicides as they require less time to reduce serious crop losses [3,4]. However, fungicides have detrimental impacts, including the emergence of fungicide-resistant pathogens, decline of soil physio–chemical properties, accumulation of toxic compounds, and long residual periods [5,6,7,8,9]. Due to increasing demand of consumers for fungicide-free products, the need for alternative disease control strategies, such as biological control has been emphasized [10,11]. Biological control agents are environment friendly and sustainable for protecting plants against pathogens [8,12,13].

The role of active metabolites derived from biocontrol agents as viable and reliable alternatives to chemical fungicides cannot be underestimated [9,14,15,16,17]. Among various biocontrol agents, *Bacillus* species show strong abilities to restrict the growth of plant pathogens by synthesizing hydrolytic enzymes and antifungal compounds [14,15,16,18,19]. Moreover, *Bacillus* species are known to produce peptide antibiotics [20,21,22] used for biocontrol of agricultural crops [23]. Peptide antibiotic compounds have received particular attention as candidates for plant protection products [24,25]. These compounds can permeate and disrupt fungal cell membranes [26,27], thus reducing the likelihood of developing resistance compared to traditional antibiotics [28]. *Bacillus velezensis* also produce peptide antibiotics that can inhibit the growth of various fungi [16,22]. Its potential utility as a biocontrol agent against several fungal plant pathogens has recently been investigated [16,29,30].

*Colletotrichum* fungi causing anthracnose disease in many economical crops worldwide, is categorized as one among the top 10 fungal pathogens [31]. These pathogens can invade host plants via melanized appressoria and spread infection by forming primary and secondary hyphae to colonize host plant cells, leading to the development of dark or water-soaked lesions with sunken necrotic tissues at infected areas. *Colletotrichum* infections are visible as lesions on leaves, fruits, and other parts of plants, resulting in yield losses and reduced crop marketability [32]. *Colletotrichum* consists of approximately 189 known species with a broad host range and high genetic diversities [33]. Although many *Colletotrichum* species are seed-borne pathogens, they can exist in soil and dead plant parts as saprophytes. Their spores can be dispersed through water splashing and by wind [33,34]. Anthracnose caused by *C. gloeosporioides* has been reported from valuable crop plants such as strawberry, dragon fruit, cassava, mango, guava, apple, coffee, avocado, almond, jujube, etc., and causes a serious economic constraint till harvest [35]. Considering the challenges posed by the disease, reliable and cost-efficient biocontrol agents are advocated. Further, the mechanisms of action of biocontrol agent-derived antibiotics against anthracnose caused by *C. gloeosporioides* remains poorly understood [36].

Numerous microorganisms have been used for controlling fungal diseases including the diseases caused by *C. gloeosporioides* [14,15,16]. However, many researchers are focusing on antagonistic microorganisms that can more effectively control *C. gloeosporioides* and improve crop production and quality. *Bacillus* strains possess the advantage of sporulation which confers heat resistance, desiccation tolerance, and the ability to successfully colonize the plant micro-environment [37,38], thereby restricting pathogen infection [39]. The objectives of this study was to isolate and identify an antifungal cyclic tetrapeptide from *B. velezensis* CE 100 and, subsequently, investigate its inhibitory effects on mycelial growth and spore germination of plant pathogen *C. gloeosporioides*.

## 2. Results

### 2.1. Antifungal Activities of B. velezensis CE 100 Culture Filtrate against Phytopathogenic Fungi

**Figure 1:**
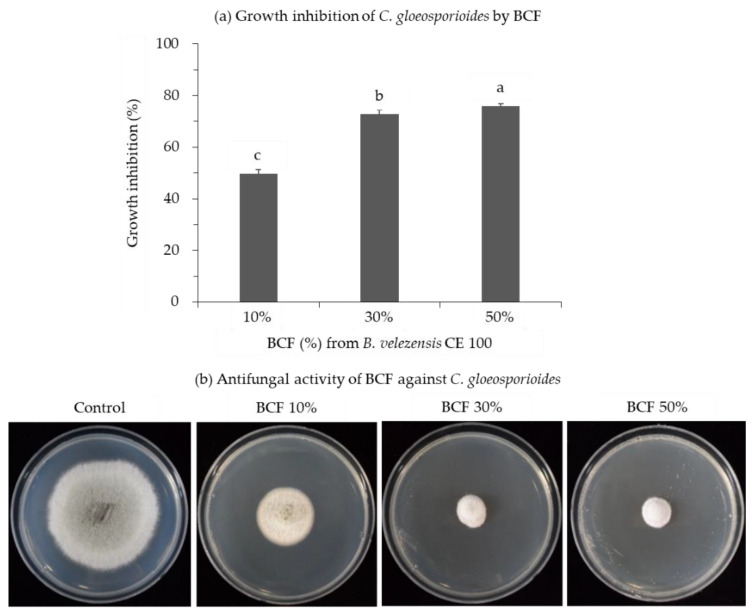
Mycelial growth inhibition of *C. gloeosporioides* by bacterial culture filtrate (BCF) from *B. velezensis* CE 100. (**a**) Growth inhibition of *C. gloeosporioides* by BCF. (**b**) Antifungal activity of BCF against *C. gloeosporioides*. Error bars represent standard deviation of the mean. Calculated mean values are from three replicates. Mean with the different letter are significantly different at *p* < 0.05 when compared using least significant difference test.

Various concentrations of *B. velezensis* CE 100 culture filtrate (BCF) were tested for antifungal properties against the plant pathogen *C. gloeosporioides* (Figure 1). BCF at all concentrations was able to inhibit the growth of the fungal pathogens. This is substantiated by the fact that the mycelial growth inhibition significantly increased with increasing concentration of BCF. BCF at a concentration of 50% showed the highest inhibition (75.9%) of mycelial growth while the inhibition was 49.7% at 10% of BCF concentration (Figure 1).

### 2.2. Isolation and Identification of Antifungal Cyclic Tetrapeptide

The antifungal compound (EE3-3) was purified from the ethyl acetate fraction of *B. velezensis* CE 100 culture broth by medium pressure liquid chromatography (MPLC) coupled with an octadecylsilane (ODS) column. The molecular weight of EE3-3 (380.2) was established by observing a sodiated molecular ion peak at *m/z* 403.2 (M+Na)^+^ in the electrospray ionization-mass spectrometry (ESI-MS) spectral analysis. ^1^H and ^13^C nuclear magnetic resonance (NMR) spectra of EE3-3 were similar to those of a cyclic tetrapeptide cyclo-(prolyl-leucyl-alanyl-isoleucyl) reported previously [40,41].

**Figure 2:**
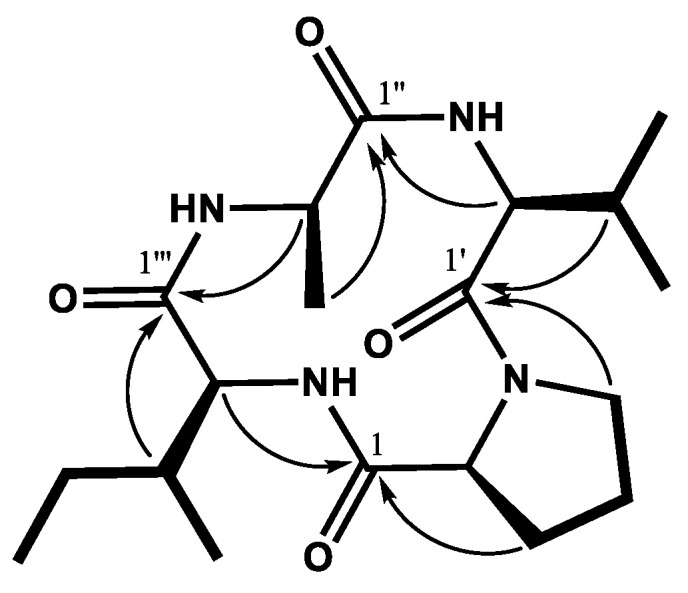
Chemical structure of the antifungal cyclic tetrapeptide [cyclo-(prolyl-valyl-alanyl-isoleucyl)] isolated from *B. velezensis* CE 100 culture broth underlying ^1^H-^1^H COSY (bold lines) and HMBC (arrows) correlations. Carbon position is represented as ′ for valine, ″ for alanine, and ‴ for isoleucine.

Further, the NMR spectral analysis identified EE3-3 to contain a valine [cyclo (prolyl valyl alanyl isoleucyl)] instead of leucine cyclo-(prolyl-leucyl-alanyl-isoleucyl). The ^13^C spectrum of EE3-3 showed the presence of 19 carbons (Table 1), including four amide carbonyl carbons at δ 172.7 (C-1), 171.4 (C-1″), 169.4 (C-1‴), and 167.7 (C-1′). The ^1^H NMR spectrum showed four methine protons at δ 4.21 (1H, t, *J* = 7.5 Hz, H-2), 4.02–4.04 (1H, m, H-2′), 4.01–4.06 (1H, m, H-2″), 3.91 (1H, d, *J* = 2.5 Hz, H-2‴), and five methyl protons at δ 0.94–1.45. Individual amino acids were assigned based on ^1^H-^1^H correlation spectroscopy (COSY; Figure 2, bold lines). Connectivities of the antifungal cyclic tetrapeptide were further confirmed by heteronuclear single quantum correlation (HSQC) and heteronuclear multiple-bond correlation (HMBC, Figure 2, arrows) experiments. The antifungal cyclic tetrapeptide was thus confirmed to be [cyclo-(prolyl-valyl-alanyl-isoleucyl)].

### 2.3. Antifungal Properties of the Cyclic Tetrapeptide against C. gloeosporioides

**Figure 3:**
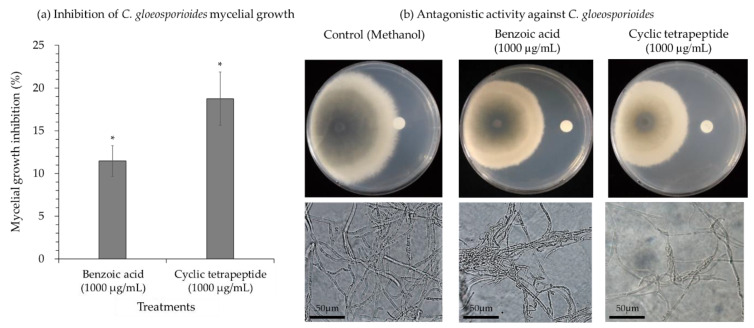
Antifungal efficacy of the cyclic tetrapeptide against *C. gloeosporioides*. (**a**) Inhibition of *C. gloeosporioides* mycelial growth. (**b**) Antagonistic activity against *C. gloeosporioides.* The experiment included methanol (as a negative control), benzoic acid derived from *B. licheniformis* MH148 (as a positive control), and the cyclic tetrapeptide from *B. velezensis* CE 100 against the fungal pathogen *C. gloeosporioides*. Error bars represent standard deviation of the mean. Calculated mean values are from three replicates. Asterisk indicates a significant difference between treatments as observed by *t*-test at *p* < 0.05.

The *C. gloeosporioides* hyphae were incubated with 1000 µg/mL of the cyclic tetrapeptide and benzoic acid (from *B. licheniformis* MH48) as a positive control. The degradation and deformation of mycelia were observed (Figure 3). The inhibition rate of the cyclic tetrapeptide on the mycelial growth of *C. gloeosporioides* was 18.8% in 1000 µg/mL, with significant differences compared to benzoic acid (11.5%) (Figure 3a). Mycelium morphology was observed under light microscopy and showed buckle formation and swelling growth in *C. gloeosporioides* hyphae in both the treatment conditions (Figure 3b).

**Figure 4:**
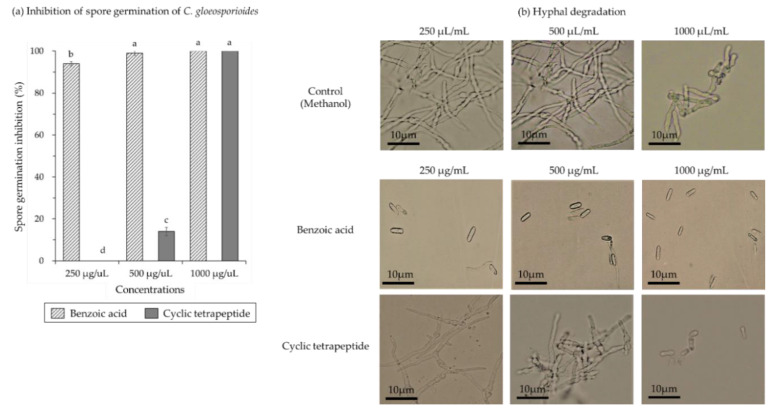
Antifungal efficacy of a cyclic tetrapeptide against spore germination of *C. gloeosporioides*. (**a**) Spore germination inhibition and (**b**) hyphal degradation of *C. gloeosporioides*. The experiment included methanol (as a negative control), benzoic acid derived from *B. licheniformis* MH148 (as a positive control), and the cyclic tetrapeptide from *B. velezensis* CE 100 against the fungal pathogen *C. gloeosporioides*. Error bars represent standard deviation of the mean. Calculated mean values are from three replicates. Mean with the different letters are significantly different at *p* < 0.05 when compared using least significant difference test.

*C. gloeosporioides* spores germination was significantly inhibited with increasing concentrations of benzoic acid and the cyclic tetrapeptide (Figure 4). The spore germination inhibition was 94.0% at 250 µg/mL of benzoic acid and complete inhibition at 1000 µg/mL (Figure 4a). The spores germinated and developed into hyphae at 250 µg/mL of cyclic tetrapeptide concentration that means no inhibition of *C. gloeosporioides* spore germination (Figure 4b). At 500 µg/mL, cyclic tetrapeptide concentration 14.0% inhibition of spore germination was observed. The *C. gloeosporioides* spores germination was totally inhibited at 1000 µg/mL of the cyclic tetrapeptide concentration (Figure 4a).

## 3. Discussion

*Bacillus* species demonstrating wide array of bioactive metabolites have been recognized as effective candidates to control several phytopathogens [14,15,16,42,43,44,45]. In this study, the BCF of *B. velezensis* CE 100 displayed significant antifungal activities against the pathogen, *C. gloeosporioides,* which causes anthracnose disease in plants. Results of in vitro tests suggested the involvement of secondary metabolites, consistent with findings from several previous studies stating the promiscuous action of the secondary metabolites against fungal pathogens [14,15,16,42,43,44,45]. In this study, we identified a cyclic tetrapeptide compound from *B. velezensis* CE 100 culture filtrate based on one- and two-dimensional NMR spectral analysis. This is the first report showing the isolation of the cyclic tetrapeptide from *B. velezensis* CE 100 culture filtrate. ^1^H and ^13^C nuclear magnetic resonance (NMR) spectra were similar to those of cyclo-(prolyl-leucyl-alanyl-isoleucyl) as reported previously [40,41]. 

Cyclic tetrapeptide caused significant antifungal activities against the fungal plant pathogen *C. gloeosporioides* revealed by the mycelial growth and spore germination results. Especially, the cyclic tetrapeptide inhibited about 1.6-fold of mycelial growth compared to that with the positive control benzoic acid. Moreover, the spore germination was totally inhibited by the action of the cyclic tetrapeptide at 1000 µg/mL. The germination of the hyphae also was inhibited by the action of the cyclic tetrapeptide. However, benzoic acid at 250 µg/mL showed strong inhibition of *C. gloeosporioides* spore germination. As reported earlier, BCF from *B. licheniformis* MH48 involves benzoic acid, which inhibits the growth of *C. gloeosporioides* by 63.1% at 50% of the BCF [14]. Further, at 50% of the BCF from *B. velezensis* CE 100 a strong antifungal activity (75.9%) was noticed against *C. gloeosporioides*. The present findings indicate that the mycelial growth and spore germination inhibition could be effective to control the plant pathogen *C. gloeosporioides*.

## 4. Materials and Methods

### 4.1. Bacterial Culture and Fungal Pathogen

The antagonistic bacterial strain *B. velezensis* CE 100 was isolated from pot soil of tomato plant [44]. The strain was streaked onto tryptone soy agar medium to obtain single colonies. Subsequently, a single colony was inoculated in tryptone soy broth (TSB) and incubated at 30 °C and 130 rpm for 2 days. The resulting cultural broth (10^7^ colony-forming unit (CFU)/mL) was mixed with 50% sterile glycerin and maintained at −80 °C for further experiments. The fungal pathogen *C. gloeosporioides* KACC 40896 used in this study was provided by Korean Agricultural Culture Collection and sub-cultured on potato dextrose agar (PDA) medium for 7 days at 25 °C.

### 4.2. Antifungal Activity of B. velezensis CE 100 Culture Filtrate 

One single colony of *B. velezensis* CE 100 was inoculated in TSB medium at 30 °C and 130 rpm for 3 days. Then 500 µL of inoculated culture broth (10^7^ CFU/mL) was inoculated again into fresh TSB medium (500 mL) at 30 °C and 130 rpm with shaking incubator for 7 days. Three replications of the inoculation were maintained. The *B. velezensis* CE 100 culture broth was centrifuged at 12,000 rpm for 15 min at 4 °C. The supernatant was collected and filtered through four layers of filter paper (Whatman No. 6). The endospore remnants in the culture filtrate were removed using a syringe filter (0.2 µm). The obtained filtrate was used for antifungal studies against *C. gloeosporioides*.

The PDA medium with different water levels (90%, 70%, and 50%) was prepared in different conical flasks, autoclaved at 121 °C for 15 min, and keep until 60 °C. Then, *B. velezensis* CE 100 culture filtrate concentrations (10%, 30%, and 50%) was added into each flask, mixed thoroughly, and poured into sterile petri dishes. PDA plates without the culture filtrate were used as controls. A mycelial plug from culture of *C. gloeosporioides* was placed at the center of the PDA plate and incubated at 25 °C for 7 days. Three replications were maintained for each assay. Mycelial growth inhibition percentage was calculated as (*R* − *r*)/*R* × 100, where *R* is the radius of the fungal colony in the control plate and *r* was the radius of the fungal colony in the treatment plate.

### 4.3. Purification and Characterization of Antifungal Compound

*B. velezensis* CE 100 was cultured in 12 L of TSB medium for 14 days at 30 °C. The culture broth was centrifuged at 6000 rpm for 30 min and the supernatant was filtered through a filter paper (Whatman No. 6). The culture filtrate was then acidified with concentrated HCl solution to pH 3 and then partitioned successively with *n*-hexane, chloroform, ethyl acetate, and water-saturated *n*-butanol (each 12 L). These fractions were concentrated with a vacuum rotary evaporator. The ethyl acetate fraction was found suitable for the inhibition of the growth of *C. gloeosporioides* by conducting paper disc method.

The antifungal compound was purified from the ethyl acetate fraction using MPLC (Isolera one, Biotage, Sweden) coupled with SNAP Ultra C18 120 g column (Isolera one, Biotage, Sweden) with gradient elution of H_2_O (A) and MeCN (B). The compounds separated were monitored at 254 and 220 nm with a flow rate of 25 and 50 mL/min. Nine fractions (EA−EI) were separated from the ethyl acetate fraction (5.0 g) by a linear gradient elution of initial 0% B for 5 min→100% B for 30 min. Fraction EE (retention time *t_R_* of 15–18 min, 632 mg) was re-eluted by a linear gradient elution of 15% B→35% B for 18 min→35% B for 28 min to obtain seven subfractions (EE1−EE7). The antifungal compound (EE3-3, *t_R_* 7.6–8.1 min, 17.2 mg) was finally purified from subfraction EE3 (*t_R_* 7.6–8.1 min, 632 mg) by linear gradient elution of 10% B for 12 min→25% B for 36 min (flow rate 25 mL/min).

The antifungal compound (EE3-3, white amorphous powder) was analyzed by MS and NMR experiments. Mass spectra were obtained on a hybrid ion-trap time-of-flight mass spectrometer (SYNAPT G2, Waters, Cambridge, UK) equipped with an electrospray ionization source at Korea Basic Science Institute (KBSI, Ochang, Cheongju, Korea). The antifungal compound was dissolved in deuterated methanol (CD_3_OD). ^1^H (500 MHz) and ^13^C (125 MHz) NMR spectra were acquired using an ^unity^INOVA 500 spectrometer (Varian, Walnut Creek, CA, USA) in Korean Basic Science Institute, Gwangju Center, Korea. The structure of compound was determined by the ^1^H−^1^H correlation spectroscopy (COSY), heteronuclear multiple-quantum coherence (HMQC) and heteronuclear multiple-bond correlation (HMBC) experiments.

### 4.4. Antifungal Properties of the Purified Compound

The purified cyclic tetrapeptide was assayed for the inhibition of the mycelial growth and spore germination against pathogen *C. gloeosporioides*. The *B. licheniformis* MH48 derived benzoic acid acted as positive control and was purchased from Daejung Chemicals, Siheung, Korea. The *C. gloeosporioides* mycelial growth inhibition by the purified cyclic tetrapeptide was assayed using the paper disc method. The purified cyclic tetrapeptide and benzoic acid were dissolved in methanol at 1000 µg/mL. A paper disc was placed on one side of the PDA plate and 50 µL from methanol (negative control), benzoic acid (positive control), and the purified cyclic tetrapeptide was loaded on the paper disc. Then, a mycelial plug (5 mm diameter) of *C. gloeosporioides* was inoculated at 4 cm distance from the paper disc on the same PDA plate. The experiment was conducted in three replications, wherein the plates were incubated at 25 °C for 7 days. The mycelial growth inhibition of *C. gloeosporioides* was measured using the following formula: mycelial growth inhibition (%) = (*M* − *m*)/*M* × 100, where *M* is the radial growth of *C. gloeosporioides* in the control plate (methanol) and *m* the radial growth of *C. gloeosporioides* in the treatment plate (benzoic acid and cyclic tetrapeptide). A small piece of mycelium from the border of *C. gloeosporioides* colony inhibited by concentrations of the purified cyclic tetrapeptide and benzoic acid was used to observe the deformation of hyphal structures under a light microscope (Olympus BX41TF, Tokyo, Japan).

The spore suspension was prepared using the *C. gloeosporioides* culture spread on PDA plates for 7 days at 25 °C. The surface of fully sporulated fungal colony was flooded with 10 mL of sterile distilled water and gently scrubbed with sterile spatula. The fungal suspension was filtered through sterile gauze to remove mycelia. The resulting spore suspension was adjusted to 1 × 10^6^ spore/mL using a hemocytometer cell-counting chamber. To measure the effects of the cyclic tetrapeptide on *C. gloeosporioides* spore germination, 2 mg of the purified cyclic tetrapeptide was first dissolved in 200 µL of methanol. Next, 2 mg of benzoic acid was dissolved in 200 µL of methanol as the positive control. Subsequently, the purified cyclic tetrapeptide and benzoic acid was serially diluted with sterile PDB medium to obtain concentrations of 250, 500, and 1000 µg/mL. Spore suspension of *C. gloeosporioides* (100 µL) was added to each vial containing different concentrations of purified cyclic tetrapeptide and benzoic acid. The vial with different concentration of methanol (without cyclic tetrapeptide and benzoic acid) was used as a negative control. Each treatment was set as three replicates. The vials were incubated at 25 °C for 10 h. A total of 100 spores from each replication of each treatment were examined using an Olympus BX41 light microscope. Numbers of germinated spores in each treatment were counted. Spore germination inhibition (%) was calculated as (*S* − *s*)/*S* × 100, where *S* and s represents the number of germinated spore in the control vials (methanol) and treatment vials (cyclic tetrapeptide and benzoic acid), respectively.

### 4.5. Statistical Analysis

To determine the differences in the mycelial growth inhibitions between benzoic acid and cyclic tetrapeptide against *C. gloeosporioides*, we used *t*-test at *p* < 0.05. The mycelial growth inhibition of the BCF concentrations and the spore germination inhibition of compounds against *C. gloeosporioides* were determined with the analysis of variance. Mean values were compared using least significant difference test at *p* < 0.05. All data were performed using SAS 9.0 software (SAS Institute, Cary, NC, USA).

## Figures and Tables

**Table 1:** ^1^H (500 MHz) and ^13^C (125 MHz) NMR data of the antifungal compound in deuterated methanol (CD_3_OD).

Residue	Position	δ_H_ (*Int.*, *Multi.*, *J* in Hz)	δ_C_
Proline	1	^a^	172.7
	2	4.21 (1H, t, 7.5)	60.2
	3	2.31−2.35 (1H, m), 1.91−1.94 (1H, m) ^b^	29.7
	4	2.03−2.06 (1H, m), 1.91−1.94 (1H, m) ^b^	23.4
	5	3.52−3.54 (2H, m)	46.3
Valine	1′	^a^	167.7
	2′	4.02−4.04 (1H, m) ^c^	61.7
	3′	2.48−2.51 (1H, m)	30.0
	4′	1.10 (3H, d, 7.0)	19.0
	5′	0.94 (3H, d, 6.5)	16.8
Alanine	1″	^a^	171.4
	2″	4.01−4.06 (1H, m) ^c^	51.7
	3″	1.45 (3H, d, 7.0)	21.1
Isoleucine	1‴	^a^	169.4
	2‴	3.91 (1H, d, 2.5)	61.1
	3‴	1.93−1.96 (1H, m)	40.4
	4‴	1.50−1.55 (1H, m), 1.23−1.29 (1H, m)	25.8
	5‴	1.03 (3H, d, 6.0)	15.7
	6‴	0.96 (3H, t, 7.5)	12.3

^a^ No proton signal. ^b^ Signals of H-3 and H-4 overlapped. ^c^ Signals of H-2′ and H-2″ overlapped. Carbon position is represented as ′ for valine, ″ for alanine, and ‴ for isoleucine.

## Data Availability

All the raw data is available and provided upon request.
